# Molecular basis for the function of the αβ heterodimer of human NAD-dependent isocitrate dehydrogenase

**DOI:** 10.1074/jbc.RA119.010099

**Published:** 2019-09-12

**Authors:** Pengkai Sun, Tengfei Ma, Tianlong Zhang, Hanwen Zhu, Jianyang Zhang, Yabing Liu, Jianping Ding

**Affiliations:** State Key Laboratory of Molecular Biology, CAS Center for Excellence in Molecular Cell Science, Institute of Biochemistry and Cell Biology, University of Chinese Academy of Sciences, Chinese Academy of Sciences, 320 Yue-Yang Road, Shanghai 200031, China

**Keywords:** allosteric regulation, conformational change, enzyme mechanism, crystal structure, enzyme structure, inhibition mechanism, tricarboxylic acid cycle (TCA cycle) (Krebs cycle), isocitrate dehydrogenase, NAD-IDH, activation

## Abstract

Mammalian mitochondrial NAD-dependent isocitrate dehydrogenase (NAD-IDH) catalyzes the decarboxylation of isocitrate into α-ketoglutarate in the tricarboxylic acid cycle. It exists as the α_2_βγ heterotetramer composed of the αβ and αγ heterodimers. Different from the αγ heterodimer that can be allosterically activated by CIT and ADP, the αβ heterodimer cannot be allosterically regulated by the activators; however, the molecular mechanism is unclear. We report here the crystal structures of the αβ heterodimer of human NAD-IDH with the α subunit in apo form and in Ca^2+^-bound, NAD-bound, and NADH-bound forms. Structural analyses and comparisons reveal that the αβ heterodimer has a similar yet more compact overall structure compared with the αγ heterodimer and contains a pseudo-allosteric site that is structurally different from the allosteric site. In particular, the β3-α3 and β12-α8 loops of the β subunit at the pseudo-allosteric site adopt significantly different conformations from those of the γ subunit at the allosteric site and hence impede the binding of the activators, explaining why the αβ heterodimer cannot be allosterically regulated by the activators. The structural data also show that NADH can compete with NAD to bind to the active site and inhibits the activity of the αβ heterodimer. These findings together with the biochemical data reveal the molecular basis for the function of the αβ heterodimer of human NAD-IDH.

## Introduction

In all aerobic organisms, the cells use the tricarboxylic acid (TCA)^2^ cycle (also known as citric acid cycle or Krebs cycle) to generate the energy in the form of ATP through the oxidation of acetyl-CoA derived from carbohydrates, fats, and proteins. Among a series of biochemical reactions in the TCA cycle, isocitrate dehydrogenases (IDHs) catalyze the oxidative decarboxylation of isocitrate (ICT) into α-ketoglutarate (α-KG) using NAD or NADP as coenzyme. Prokaryotic cells contain only NADP-dependent IDHs (NADP-IDHs) in the cytosol to catalyze the reaction in the TCA cycle. However, eukaryotic cells contain both NADP-IDHs and NAD-dependent IDHs (NAD-IDHs); the NAD-IDHs localized in the mitochondria catalyze the reaction in the TCA cycle, and the NADP-IDHs localized in the cytosol and mitochondria play important roles in cellular defense against oxidative damage, detoxification of reactive oxygen species, and synthesis of fat and cholesterol (1–4).

In human cells, mutations of cytosolic and mitochondrial NADP-IDHs (also called IDH1 and IDH2) have been identified in multiple types of tumors, and the mutant proteins confer a new function to convert α-KG into 2-hydroxyglutarate, the accumulation of which can result in altered metabolism and epigenetic dysregulation of gene expression leading to pathogenesis and progression of cancers (5–9). Recently, abnormal expression or mutations of human NAD-IDH (also called IDH3) are also found to be associated with the development of cancers and diseases. Aberrant expression of the α subunit of NAD-IDH (IDH3α) is found in glioblastoma and bipolar disorder patients, and elevated expression can promote progression of glioblastoma and other malignant tumors through regulation of one-carbon metabolism or HIF-1–mediated metabolic reprogramming and angiogenesis (10–13). A variety of mutations in IDH3α have been identified in patients who exhibited childhood onset of neurological defects (14) and retinal degeneration (15). In mice, IDH3α mutations can cause retinal degeneration and reduced mitochondrial function (16). In addition, homozygous mutations in IDH3β have been identified in patients with familial nonsyndromic retinal degeneration; however, none of those patients exhibited symptoms of mitochondrial dysfunction other than retinitis pigmentosa (17). Moreover, elevated human NAD-IDH activity can cause mitochondrial Ca^2+^ uptake and reactive oxygen species production, leading to apoptosis of alveolar epithelial cells type 2, and thus is also implicated in the pathogenesis of adult respiratory distress syndrome (18). Therefore, the functional, structural, and mechanistic studies of both types of IDHs have important biological and biomedical significance.

The structure, function, and catalytic mechanism of NADP-IDHs from both prokaryotes and eukaryotes have been extensively studied (19–23). These enzymes exist and function as homodimers, which employ a conserved catalytic mechanism but different regulatory mechanisms. *Escherichia coli* and possibly other prokaryotic NADP-IDHs regulate their activity through reversible phosphorylation of a strictly conserved Ser residue at the active site (24, 25). Human cytosolic and probably other eukaryotic NADP-IDHs are likely to regulate their activity through substrate binding–induced conformational changes of the active site (22).

Eukaryotic NAD-IDHs are more complex than NADP-IDHs in both composition and regulation. Yeast NAD-IDH consists of a catalytic subunit and a regulatory subunit that form a heterodimer, which is assembled into a heterotetramer and further into a heterooctamer (26–31). Different from yeast NAD-IDH, human and other mammalian NAD-IDHs are composed of three types of subunits in a ratio of 2α:1β:1γ (32–38). The α and β subunits form a heterodimer (αβ), and the α and γ subunits form another heterodimer (αγ), which are assembled into a heterotetramer (α_2_βγ) and further into a heterooctamer (the heterotetramer and heterooctamer are also called holoenzyme). Early biochemical studies showed that the enzymatic activity of mammalian NAD-IDHs could be activated by CIT and ADP; and in the holoenzyme, the α subunit exerts the catalytic activity, and the β and γ subunits play the regulatory roles (32–38). Our biochemical studies of human NAD-IDH further showed that the αγ heterodimer can be allosterically activated by CIT and ADP, whereas the αβ heterodimer cannot, and both heterodimers can be inhibited by NADH (39). Our structural and kinetic studies of the αγ heterodimer of human NAD-IDH revealed that the binding of CIT and ADP to the allosteric site in the γ subunit induces conformational changes at the allosteric site, which are transmitted to the active site in the α subunit via the heterodimer interface, leading to the decrease of the *S*_0.5,ICT_ and thus the activation of the enzyme (40). NADH can bind to the allosteric site to compete with the binding of the activators and meanwhile to the active site to compete with the binding of NAD, leading to the inhibition of the enzyme (41). However, so far, the molecular basis for the function of the αβ heterodimer is still unclear.

In this work, we determined the crystal structures of the αβ heterodimer of human NAD-IDH with the α subunit in apo form and in Ca^2+^-bound, NAD-bound, and NADH-bound forms. Structural analyses and comparisons reveal that the αβ heterodimer assumes a more compact overall conformation than the αγ heterodimer and contains a pseudo-allosteric site that is structurally different from the allosteric site and thus is unable to bind the activators. Due to the conformational changes of the heterodimer interface, the active site has a distorted geometry that is unable to bind the metal ion effectively or in a catalysis-relevant manner. NADH can compete with NAD to bind to the active site and hence inhibits the activity of the enzyme. These findings together with the biochemical data provide the molecular basis for why the αβ heterodimer alone cannot be activated by CIT and ADP but can be inhibited by NADH and might explain why the αβ heterodimer alone has a high *S*_0.5,Mn_ and a low activity.

## Results

### Preparation and biochemical analysis of the αβ heterodimer of human NAD-IDH

The WT and mutant αβ heterodimers of human NAD-IDH were prepared as described previously (39). The WT αβ heterodimer could yield crystals at multiple crystallization conditions, which, however, diffract X-rays very poorly (about 8–10 Å). Sequence analysis shows that the C-terminal regions of the α, β, and γ subunits of human NAD-IDH are substantially different from each other and also varied among different species (Fig. S1). After various trials, we obtained a stable mutant αβ heterodimer in which the C-terminal region (residues 341–349) of the β subunit was substituted with the corresponding region (residues 330–338) of the α subunit, and this mutant led to the successful determination of the crystal structures of the αβ heterodimer. Like the WT αβ heterodimer, the mutant αβ heterodimer exists as a heterodimer in solution with high purity and homogeneity, as shown by size-exclusion chromatography and SDS-PAGE analyses (Fig. S2).

**Figure 1.**
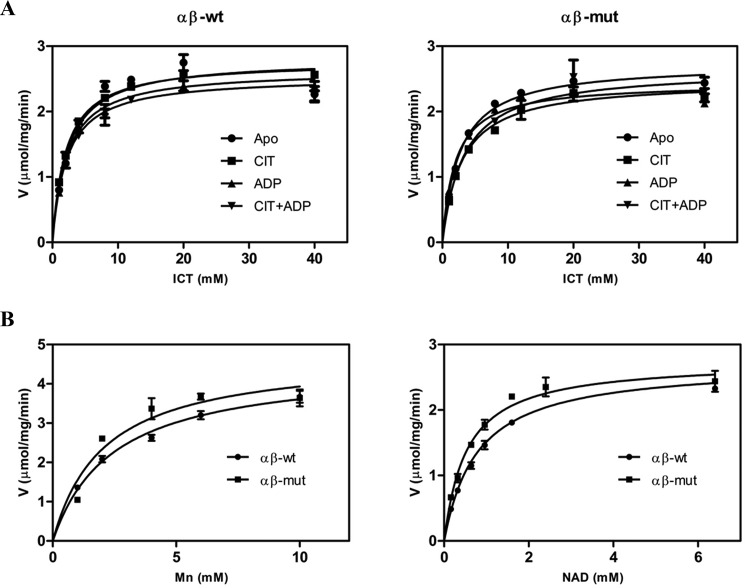
**Saturation curves of the WT and mutant αβ heterodimers.** *A*, ICT saturation curves of the WT αβ (*left*) and mutant αβ (*right*) in the absence and presence of the activator CIT and/or ADP. *B*, Mn^2+^ saturation curves of the WT αβ and mutant αβ (*left*) and NAD saturation curves of the WT αβ and mutant αβ (*right*). The activities were measured at standard conditions with varied concentrations of the substrate ICT, the metal ion Mn^2+^, or the cofactor NAD, respectively. The values are the averages of two independent measurements with the S.E. (*error bars*).

In our previous biochemical studies, we found that the αβ heterodimer has a weaker activity than the αγ heterodimer at standard conditions (39). As the αβ heterodimer exhibits a significantly higher *S*_0.5,Mn_, the kinetic data of the αβ heterodimer were measured at a much higher concentration of MnCl_2_ (50 mm instead of 2 mm). To make the kinetic data of the αβ heterodimer, the αγ heterodimer, and the holoenzyme comparable, in this work, we remeasured the kinetic data of the αβ heterodimer at standard conditions, albeit with slightly higher deviations. The αβ heterodimer exhibits a specific activity of 2.80 ± 0.14 μmol/min/mg, an *S*_0.5,ICT_ of 2.29 ± 0.45 mm, an *S*_0.5,Mn_ of 2.38 ± 0.27 mm, and an *S*_0.5,NAD_ of 0.791 ± 0.081 mm (Table 1 and Fig. 1), which are comparable with those reported in our previous work measured at high concentration of MnCl_2_ (39). In the presence of CIT or/and ADP, the αβ heterodimer exhibits similar *V*_max_ and *S*_0.5,ICT_ as in the absence of the activators, confirming that the αβ heterodimer cannot be activated by CIT and/or ADP (Table 1 and Fig. 1). Furthermore, the mutant αβ heterodimer exhibits almost identical activity and kinetic parameters as the WT enzyme, indicating that the substitution of the C-terminal region of the β subunit has no effect on the enzymatic properties of the αβ heterodimer (Table 1 and Fig. 1). Therefore, we will not distinguish the WT and mutant αβ heterodimers hereafter.

**Table 1:** **Enzymatic activity and kinetic parameters of the αβ heterodimer**

Enzyme	Apo					+CIT		+ADP		+CIT+ADP		Reference
	*V*_max,ICT_*^a^*	*S*_0.5,ICT_	*k*_cat_	*S*_0.5,Mn_	*S*_0.5,NAD_	*V*_max_,_ICT_	*S*_0.5,ICT_	*V*_max,ICT_	*S*_0.5,ICT_	*V*_max,ICT_	*S*_0.5,ICT_	
	μ*mol/mg/min*	*mm*	*s*^−*1*^	μ*m*	μ*m*	μ*mol/mg/min*	*mm*	μ*mol/mg/min*	*mm*	μ*mol/mg/min*	*mm*	
α_2_βγ	20.0 ± 0.1	2.35 ± 0.05	26.7 ± 0.1	60.2 ± 6.0	143 ± 5	20.7 ± 0.3	1.27 ± 0.06	22.1 ± 0.3	0.87 ± 0.02	21.3 ± 0.4	0.16 ± 0.01	Ref. 39
αγ	7.29 ± 0.11	4.49 ± 0.15	9.72 ± 0.15	95.1 ± 3.2	238 ± 18	10.0 ± 0.2	2.61 ± 0.12	9.42 ± 0.09	1.69 ± 0.05	13.1 ± 0.4	0.18 ± 0.02	Ref. 39
αβ	2.80 ± 0.14	2.29 ± 0.45	3.73 ± 0.19	2384 ± 273	791 ± 81	2.77 ± 0.03	2.05 ± 0.10	2.64 ± 0.07	2.19 ± 0.24	2.54 ± 0.08	2.16 ± 0.29	This work
αβ mut	2.73 ± 0.05	2.64 ± 0.19	3.64 ± 0.07	2067 ± 612	540 ± 100	2.47 ± 0.07	2.94 ± 0.31	2.44 ± 0.07	1.97 ± 0.24	2.65 ± 0.11	3.29 ± 0.52	This work

*^a^* The enzymatic activity and kinetic data of the αβ heterodimer were measured at standard conditions with varied concentrations of substrate ICT, metal Mn^2+^, or co-factor NAD, respectively. A molecular mass of 80 kDa was used to calculate the moles of the αβ heterodimer per mg of protein (equivalent to 1.25×10^−8^ mol of enzyme/mg of protein). For comparison, the activities and kinetic parameters of the αγ heterodimer and the α_2_βγ heterotetramer are also listed.

### Crystal structures of the αβ heterodimer in different forms

We could obtain crystals of the αβ heterodimer at two different crystallization conditions. Crystals of the αβ heterodimer with the α subunit in apo form (αβ) grew at condition I and belong to space group *C*2, and the structure was determined at 3.06 Å resolution with each asymmetric unit containing eight αβ molecules, which form four dimers of heterodimers via the clasp domains (Table 2). In this structure, there is no metal ion or ligand bound at the active site.

**Table 2:** **Crystallographic diffraction data and refinement statistics** Values shown in parentheses are for the highest-resolution shell.

	αβ	α^Ca^β	α^NAD^β	α^NADH^β
**PDB code**	6KDF	6KDE	6KDY	6KE3
**Diffraction data**				
Wavelength (Å)	0.9789	0.9793	0.9792	0.9792
Space group	*C*2	*I*4	*P*2_1_	*P*2_1_
Cell parameters				
*a* (Å)	208.91	166.20	99.46	99.37
*b* (Å)	170.43	166.20	162.98	162.67
*c* (Å)	208.09	128.13	114.41	114.78
α (degrees)	90	90	90	90
β (degrees)	103.43	90	100.31	100.38
γ (degrees)	90	90	90	90
Resolution (Å)	50.0–3.06 (3.17–3.06)	50.0–3.00 (3.11–3.00)	50–3.02 (3.13–3.02)	50–3.30 (3.42–3.30)
Observed reflections	918,907	131,532	240,787	211,050
Unique reflections (*I*/σ(*I*) > 0)	134,178	34,725	71,108	48,488
Average redundancy	6.8 (6.3)	3.8 (3.6)	3.4 (3.4)	4.4 (4.6)
Average *I*/σ(*I*)	15.2 (2.0)	16.1 (2.1)	17.1 (2.2)	12.2 (1.6)
Completeness (%)	99.9 (99.9)	99.4 (99.4)	99.8 (99.9)	91.3 (89.9)
*R*_merge_ (%)	13.4 (104.0)	6.3 (50.4)	8.0 (50.5)	11.3 (54.1)
CC_½_ (%)	99.6 (62.8)	99.6 (83.6)	98.7 (78.5)	99.8 (78.4)
**Refinement and structure model**				
No. of reflections (*F_o_* > 0σ(*F_o_*))	131,272	34,690	71,060	47,744
Working set	124,679	32,984	67,462	45,452
Test set	6,593	1,706	3,598	2,292
*R*_work_/*R*_free_ factor (%)	17.7/22.7	19.4/24.3	19.7/24.6	25.0/29.9
Total protein atoms	39,059	9,630	19,885	19,231
Total metal atoms		2		
Total ligand atoms			271	176
Molecules/asymmetric unit	8	2	4	4
Wilson B factor (Å^2^)	65.1	78.0	69.3	60.4
Average B factor (Å^2^)	55.2	75.2	71.4	60.0
Protein atoms	55.2	75.2	71.3	59.8
Metal atoms		70.1		
Ligand atoms			79.4	79.3
Root mean square deviations				
Bond lengths (Å)	0.010	0.009	0.010	0.013
Bond angles (degrees)	1.2	1.1	1.1	1.4
Ramachandran plot (%)				
Most favored	93.2	92.9	93.7	86.0
Allowed	6.8	7.1	6.3	14.0
Disallowed	0	0	0	0

Crystals of the αβ heterodimer with the α subunit bound with a Ca^2+^ at the active site (α^Ca^β) grew at condition II and belong to space group *I*4, and the structure was determined at 3.0 Å resolution with each asymmetric unit containing two α^Ca^β molecules, which form a dimer of heterodimers via the clasp domains (Table 2). In this structure, there is evident electron density at the active site, which is interpreted as a Ca^2+^ due to the presence of 0.2 m Ca^2+^ in the crystallization solution and a reasonable *B* factor after structure refinement (Fig. S3*A*). We also obtained crystals of the α^Ca^β heterodimer at condition II in the presence of CIT or/and ADP (both 50 mm); however, in these structures, there is only a Ca^2+^ bound at the active site and no CIT or ADP bound in either the α or β subunit.

Crystals of the αβ heterodimer with the α subunit bound with an NAD at the active site (α^NAD^β) grew at condition II with the protein solution incubated with ICT and NAD (both 50 mm) before crystallization and belong to space group *P*2_1_, and the structure was determined at 3.02 Å resolution with each asymmetric unit containing four α^NAD^β molecules, which form two dimers of heterodimers via the clasp domains (Table 2). In this structure, there is clear electron density for an NAD but no electron density for Ca^2+^ or ICT at the active site (Fig. S3*B*).

Crystals of the αβ heterodimer with the α subunit bound with an NADH at the active site (α^NADH^β) also grew at condition II with the protein solution incubated with α-KG and NADH (both 50 mm) before crystallization and belong to space group *P*2_1_, and the structure was determined at 3.30 Å resolution with each asymmetric unit containing four α^NADH^β molecules, which form two dimers of heterodimers via the clasp domains (Table 2). In this structure, there is clear electron density for an NADH but no electron density for Ca^2+^ or α-KG at the active site (Fig. S3*C*).

**Figure 2.**
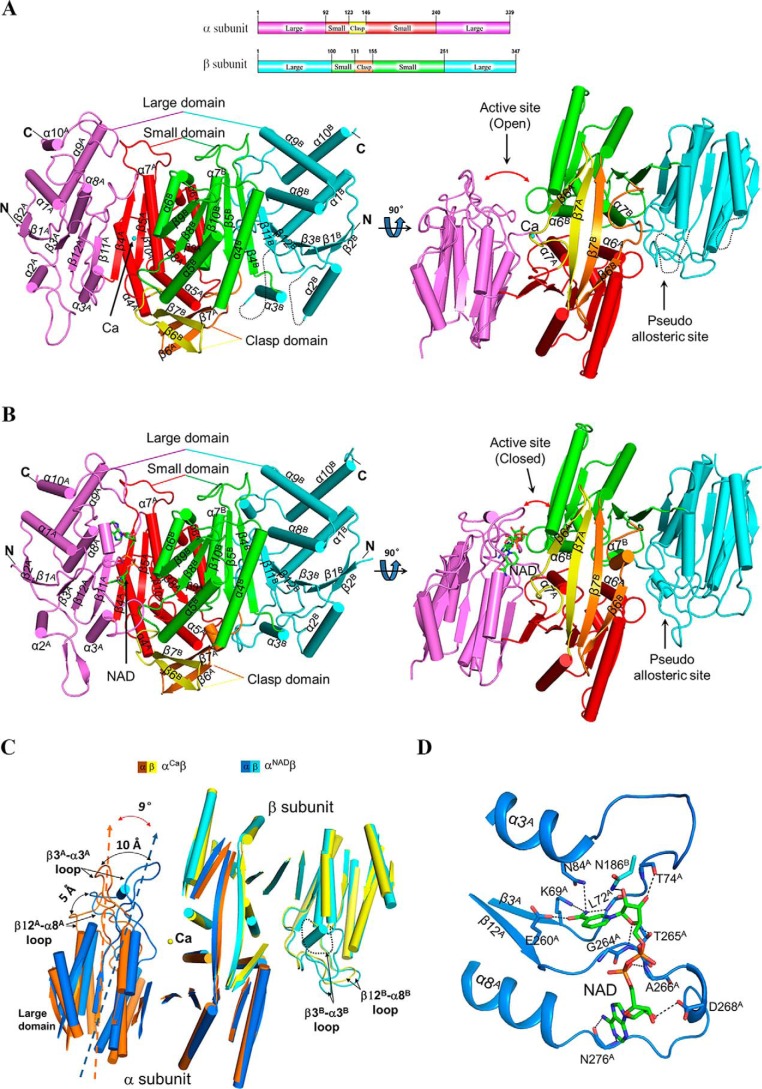
**Overall structure of the αβ heterodimer of human NAD-IDH.** *A*, overall structure of α^Ca^β in two different orientations. *Right*, view along the pseudo-2-fold axis of the αβ heterodimer. *Left*, view perpendicular to the pseudo-2-fold axis of the αβ heterodimer. The *color-coding scheme* of individual domains of the α and β subunits is shown *above*. The bound Ca^2+^ is shown as a *sphere*. Secondary structure elements of the α and β subunits are *labeled* with *superscripts A* and *B*, respectively. The disordered regions are shown with *black dotted lines. B*, overall structure of α^NAD^β in two different orientations. The *color-coding scheme* is the same as in Fig. 2*A*. The bound NAD is shown with a *ball-and-stick model. C*, comparison of α^Ca^β and α^NAD^β based on superposition of the β subunits. The *color-coding scheme* of the α and β subunits of α^Ca^β and α^NAD^β is shown *above*. Upon the NAD binding, the large domain of the α subunit exhibits a notable rotation toward the small domain of the β subunit, and particularly the β3^A^-α3^A^ loop and the β12^A^-α8^A^ loop move toward the β subunit by about 10 and 5 Å, respectively. For clarity, only the α-helices and β-strands are shown, and the loops are omitted except for the β12-α8 and β3-α3 loops. *D*, interactions of NAD with the surrounding residues in α^NAD^β. The NAD is shown with a *ball-and-stick model*, and the residues involved in the interactions are shown with side chains and *labeled*. Hydrogen-bonding interactions are indicated with *black dashed lines*.

Similar to the αγ heterodimer, in the structures of the αβ heterodimer, both the α and β subunits consist of 10 α-helices and 12 β-strands, which fold into a large domain, a small domain, and a clasp domain (Fig. 2). The α and β subunits form a heterodimer with a pseudo-2-fold symmetry, and the heterodimer interface is mediated mainly via the α6 and α7 helices of the small domains and the β6 and β7 strands of the clasp domains. The active site is located in the cleft formed by the large and small domains of the α subunit and the small domain of the β subunit. There is a pseudo-allosteric site located in the cleft formed by the large and small domains of the β subunit and the small domain of the α subunit (see below). Of note, in all of the αβ structures, the C-terminal region of the β subunit is located on the structure surface and not involved in crystal packing or ligand binding, consistent with the biochemical data showing that the mutant αβ heterodimer exhibits enzymatic properties almost identical to those of the WT enzyme.

In the αβ and α^Ca^β structures, most residues of the two subunits are well-defined except the β2-α2 loop (residues 52–60) and the β3-α3 loop (residues 80–93) of the β subunit and a few residues at the N and C termini of the α and β subunits (Fig. 2*A*). In the α^NAD^β and α^NADH^β structures, most residues of the α and β subunits, including the β2-α2 and β3-α3 loops of the β subunit, are well-defined except a few residues at the N and C termini of the α and β subunits (Fig. 2*B*). In all of the αγ structures, the two corresponding loops are disordered in the α subunit but well-defined in the γ subunit (40, 41). In all of the αγ and αβ structures, the β2-α2 loops of the α, β, and γ subunits are exposed on the structure surface but are not involved in crystal packing, suggesting that they have intrinsic flexible conformations. As the β3-α3 loops of the α, β, and γ subunits (residues 71^A^–82^A^, 80^B^–93^B^, and 78^G^–91^G^; residues and secondary structure elements of the α, β, and γ subunits are indicated with superscript “A”, “B”, and “G”, respectively) form part of the active site, the pseudo-allosteric site, and the allosteric site, respectively, the conformational differences of these loops in the three subunits are correlated with their differed functions (see below).

### The NAD binding induces significant conformational changes of the α subunit

Structural comparison shows that the four αβ structures can be divided into two groups. The αβ and α^Ca^β structures belong to one group, which share a similar overall structure with an RMSD of about 0.7 Å for about 600 Cα atoms, and the α^NAD^β and α^NADH^β structures belong to the other, which share a similar overall structure with an RMSD of about 0.7 Å for about 630 Cα atoms, whereas superposition of the αβ structures in group I (α^Ca^β) and group II (α^NAD^β) yields an RMSD of about 1.6 Å for about 570 Cα atoms. As the α^Ca^β and α^NAD^β structures have relatively higher resolutions, more complete structure models, and better quality of electron density, they will be used as representatives of the two groups in the structure analysis and comparison unless otherwise specified.

Comparison of the α^Ca^β and α^NAD^β structures shows that upon the NAD binding, the β subunit exhibits no major conformational change (an RMSD of <0.8 Å for about 320 Cα atoms), whereas the large domain of the α subunit exhibits a notable rotation (about 9°) toward the heterodimer interface compared with that in the α^Ca^β structure, and particularly the β3^A^-α3^A^ and β12^A^-α8^A^ loops undergo substantial conformational changes, moving toward the heterodimer interface by about 10 and 5 Å, respectively, yielding a “closed” active site (Fig. 2*C*). As a result, several key residues on the β3^A^-α3^A^ and β12^A^-α8^A^ loops are in proper positions and/or conformations to interact with NAD. Similar conformational changes are observed in NADP-IDHs, including human cytosolic NADP-IDH, *Saccharomyces cerevisiae* mitochondrial NADP-IDH, and *E. coli* NADP-IDH, which induce the key residues at the active site to adopt proper positions and/or conformations to interact with the metal ion, substrate, and cofactor (22, 23, 42).

Like in the structures of NADP-IDHs, in the α^NAD^β structure, the cofactor NAD is bound at the deep pocket of the active site; the nicotinamide moiety is positioned in close to the ICT-binding site, and the adenine moiety is located at the far end of the active site. A number of strictly conserved residues from the large domain of the α subunit, including Lys-69^A^, Leu-72^A^, Thr-74^A^, and Asn-84^A^ of the β3^A^-α3^A^ loop and Glu-260^A^, Gly-264^A^, Thr-265^A^, Ala-266^A^, Asp-268^A^, and Asn-276^A^ of the β12^A^-α8^A^ loop, are involved in the binding of NAD via hydrogen-bonding interactions (Fig. 2*D*). In particular, the ribose 2′-OH of the adenosine moiety, which distinguishes NAD from NADP, is recognized by the side chain of Asp-268^A^ via a hydrogen-bonding interaction, confirming the notion that Asp-268^A^ of human NAD-IDH (or the equivalents of other NAD-IDHs) is the specificity determinant for NAD against NADP (41).

### The αβ heterodimer contains a pseudo-allosteric site that is unable to bind the activators

**Figure 3.**
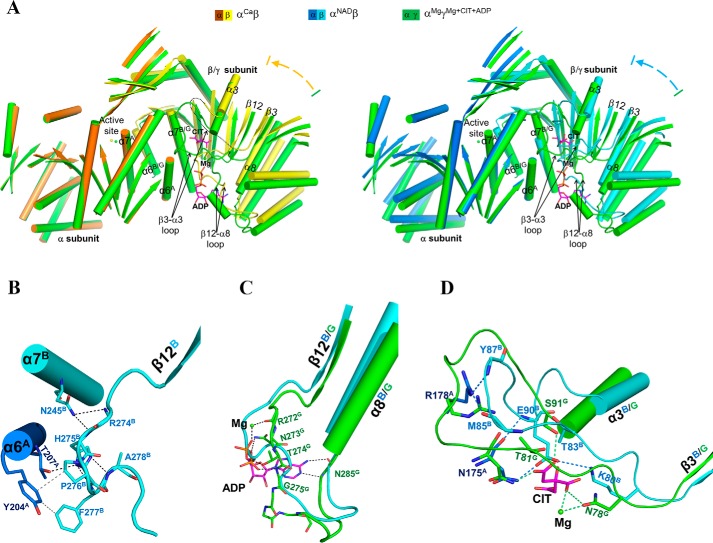
**The αβ heterodimer adopts a compact overall conformation and contains a pseudo-allosteric site.** *A*, *left*, comparison of α^Ca^β and α^Mg^γ^Mg+CIT+ADP^ (*green*) based on superposition of the α subunits. *Right*, comparison of α^NAD^β and α^Mg^γ^Mg+CIT+ADP^ (*green*) based on superposition of the small domains of the α subunits. The *color-coding scheme* of the α^Ca^β, α^NAD^β, and α^Mg^γ^Mg+CIT+ADP^ structures is shown *above*. For clarity, only the α-helices and β-strands are shown, and the loops are omitted except for the β12-α8 and β3-α3 loops of the β and γ subunits. The bound Mg^2+^, CIT, and ADP in α^Mg^γ^Mg+CIT+ADP^ are shown as a *green sphere* and *purple ball-and-stick models*, respectively. The β subunit exhibits a notable rotation toward the α subunit compared with the γ subunit, and the αβ heterodimer adopts a more compact overall conformation than the αγ heterodimer. *B*, interactions between the β12^B^-α8^B^ loop and the α6^A^ and α7^B^ helices in α^NAD^β. Hydrogen-bonding interactions are indicated with *black dotted lines*, and hydrophobic interactions are indicated with *gray dotted lines. C*, conformational difference of the β12-α8 loop (or the ADP-binding site) between α^NAD^β and α^Mg^γ^Mg+CIT+ADP^. Hydrogen-bonding interactions between ADP (and Mg^2+^) and the surrounding residues in α^Mg^γ^Mg+CIT+ADP^ are indicated with *green dotted lines. D*, conformational difference of the β3-α3 loop (or the CIT-binding site) between α^NAD^β and α^Mg^γ^Mg+CIT+ADP^. Hydrogen-bonding interactions between CIT (and Mg^2+^) and the surrounding residues in α^Mg^γ^Mg+CIT+ADP^ are indicated with *green dotted lines*, and those between the β3-α3 loop and the surrounding residues in α^NAD^β are shown with *cyan dotted lines*.

Our biochemical data show that the αβ heterodimer cannot be activated by CIT and/or ADP (Table 1), and our crystallization experiments also show that CIT and/or ADP cannot bind to the αβ heterodimer (see above). To understand the underlying molecular mechanism, we performed comparisons of the α^Ca^β and α^NAD^β structures with the α^Mg^γ^Mg+CIT+ADP^ structure, which reveal substantial conformational differences between the β and γ subunits. The α subunits in the α^Ca^β and α^Mg^γ^Mg+CIT+ADP^ structures assume very similar conformation and could be superimposed very well with an RMSD of 0.6 Å for about 320 Cα atoms, whereas the β and γ subunits are poorly superimposed with an RMSD of 1.6 Å for 320 Cα atoms. When the α subunits in the two structures are superimposed together, the β subunit exhibits a notable rotation toward the α subunit with the structure elements pulled closer to the α subunit with varied degrees of movements (about 1.3–3.2 Å) compared with the γ subunit (Fig. 3*A*, *left*). As the NAD binding induces the conformational changes of the large domain of the α subunit, the comparison of the α^NAD^β and α^Mg^γ^Mg+CIT+ADP^ structures was based on the superposition of the small domains of the α subunits, which also reveals a similar rotation of the β subunit toward the α subunit (Fig. 3*A*, *right*). As a result, the αβ heterodimer assumes a more compact overall structure than the αγ heterodimer.

Detailed structural comparisons reveal that the β3^B^-α3^B^ and β12^B^-α8^B^ loops exhibit significant conformational changes from the β3^G^-α3^G^ and β12^G^-α8^G^ loops (Fig. 3*A*). In the α^Mg^γ^Mg+CIT+ADP^ structure, the β3^G^-α3^G^ and β12^G^-α8^G^ loops form part of the allosteric site and are involved in the binding of ADP and CIT. Specifically, Arg-272^G^ of the β12^G^-α8^G^ loop is involved in the binding of CIT, and Asn-273^G^, Thr-274^G^, and Gly-275^G^ are involved in the binding of ADP (40). However, in the α^Ca^β (and other αβ) structures, the β12^B^-α8^B^ loop moves closer toward the heterodimer interface, and the equivalent residues Arg-274^B^, His-275^B^, Pro-276^B^, and Phe-277^B^ from this loop make hydrogen-bonding and hydrophobic interactions among themselves or with residues of the α7^B^ and α6^A^ helices to stabilize its conformation (Fig. 3*B*). With this conformation, the β12^B^-α8^B^ loop occupies part of the ADP-binding site in the α^Mg^γ^Mg+CIT+ADP^ structure and hence impedes the ADP binding (Fig. 3*C*).

In the α^Mg^γ^Mg+CIT+ADP^ structure, the β3^G^-α3^G^ loop adopts a wide U-shape loop conformation, and several residues of this loop, including Asn-78^G^, Thr-81^G^, and Ser-91^G^, form several hydrogen bonds with CIT and a coordination bond with Mg^2+^ at the allosteric site (40). Compared with the α^Mg^γ structure, the CIT and ADP binding induces no major conformational change of the β3^G^-α3^G^ loop except that the side chains of those residues are slightly pulled closer to interact with CIT (40). However, in the αβ and α^Ca^β structures, the β3^B^-α3^B^ loop is disordered, whereas in the α^NAD^β and α^NADH^β structures, the β3^B^-α3^B^ loop assumes a narrow U-shape loop conformation substantially different from the β3^G^-α3^G^ loop in the α^Mg^γ^Mg+CIT+ADP^ structure, and several residues on the loop form hydrogen-bonding interactions among themselves or with the surrounding residues to stabilize its conformation (Fig. 3*D*). With this conformation, the β3^B^-α3^B^ loop occupies part of the CIT-binding site in the α^Mg^γ^Mg+CIT+ADP^ structure and hence blocks the CIT binding (Fig. 3*D*).

Sequence analysis of NAD-IDHs from representative vertebrates show that the residues composing the β3-α3 and β12-α8 loops are highly conserved in the α, β, and γ subunits among different species, respectively, but are largely different from each other (Fig. S1). Particularly, the key residues of the γ subunit involved in the CIT and ADP binding are substantially different from those of the β subunit. These results together demonstrate that the β and γ subunits exhibit substantial conformational differences, and particularly the unique conformations of the β3^B^-α3^B^ and β12^B^-α8^B^ loops render the αβ heterodimer a pseudo-allosteric site that is unable to bind CIT and ADP. This explains why the αβ heterodimer cannot be allosterically regulated by the activators.

### The α^Ca^β structure assumes an active overall conformation similar to that in the α^Mg^γ^CIT+Mg+ADP^ structure

**Figure 4.**
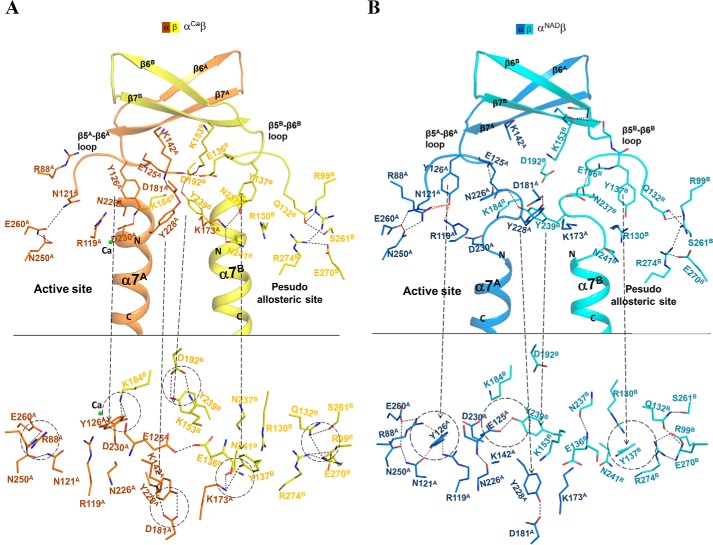
**The αβ heterodimer exhibits two different overall conformations.** *A*, a *zoom-in view* of α^Ca^β, which assumes an active overall conformation showing the conformations and interactions of key residues at the pseudo-allosteric site, the heterodimer interface, and the active site. The *color-coding scheme* of the α and β subunits of α^Ca^β is shown *above. Top*, view perpendicular to the pseudo-2-fold axis of the αβ heterodimer. *Bottom*, view along the pseudo-2-fold axis of the αβ heterodimer. *B*, a *zoom-in view* of α^NAD^β, which assumes an inactive overall conformation showing the conformations and interactions of the key residues at the pseudo-allosteric site, the heterodimer interface, and the active site. The *color-coding scheme* of the α and β subunits of α^NAD^β is shown *above*.

Structural analysis shows that in the α^Ca^β structure, the N-terminal region of helix α7 in both the α and β subunits assumes the active α-helical conformation, and the key residues Tyr-126^A^ and Lys-142^A^ at the active site and Tyr-137^B^ and Lys-153^B^ at the pseudo-allosteric site (equivalent to Tyr-135^G^ and Lys-151^G^ at the allosteric site) also assume active conformations similar to those in the α^Mg^γ^Mg+CIT+ADP^ heterodimer (40) (Fig. 4*A*). In other words, the α^Ca^β structure exhibits an active overall conformation. A detailed analysis of the α^Ca^β structure reveals the underlying mechanism.

In the αβ heterodimer, two key residues equivalent to Asn-130^G^ and Asn-259^G^ at the allosteric site are substituted with Gln-132^B^ and Ser-261^B^, respectively (Fig. S1). In the α^Ca^β structure, the longer side chain of Gln-132^B^ pushes the side chain of Tyr-137^B^ away to adopt the active conformation, which is stabilized by a hydrogen bond with the side chain of Lys-173^A^. This hydrogen-bonding interaction also induces the β5^B^-β6^B^ loop to assume the active conformation, which further triggers the conformational changes of the structure elements at the heterodimer interface and the active site, including the N-terminal region of helix α7 in both the α and β subunits and the β5^A^-β6^A^ loop at the active site. Concurrently, a number of conserved residues form a hydrogen-bonding network to stabilize the conformations of those structure elements in a similar manner as that in the α^Mg^γ^CIT+Mg+ADP^ structure (40) (Fig. 4*A*). These results suggest that the active overall conformation of the α^Ca^β structure could be attributed to the changes of two key residues at the pseudo-allosteric site, which stabilize the side chain of Tyr-137^B^ and the β5^B^-β6^B^ loop in the active conformations. In the αβ structure, the key residues at the pseudo-allosteric site, the heterodimer interface, and the active site assume similar conformations and form hydrogen-bonding interactions comparable with those in the α^Ca^β structure, indicating that the αβ structure also exhibits an active overall conformation (data not shown).

### The α^NAD^β structure assumes an inactive overall conformation similar to that in the α^Mg^γ structure

On the other hand, structural analysis shows that in the α^NAD^β structure, the N-terminal region of helix α7 in both the α and β subunits assumes the inactive loop conformation, and the key residues Tyr-126^A^ and Lys-142^A^ at the active site and Tyr-137^B^ and Lys-153^B^ at the pseudo-allosteric site also assume the inactive conformations as those in the α^Mg^γ structure (Fig. 4*B*). In other words, the α^NAD^β structure assumes an inactive overall conformation. A detailed analysis of the α^NAD^β structure reveals the underlying mechanism.

Compared with the α^Ca^β structure, the NAD binding induces the conformational changes of the β3^A^-α3^A^ loop and the β12^A^-α8^A^ loop, yielding a closed active site. With these conformational changes, a number of key residues of the β3^A^-α3^A^ and β12^A^-α8^A^ loops are in proper positions to interact with NAD. In addition, the side chain of Arg-88^A^ on the β3^A^-α3^A^ loop forms a hydrogen bond with the side chain of Tyr-126^A^ on the β5^A^-β6^A^ loop, which induces the side chain of Tyr-126^A^ to assume the inactive conformation that is further stabilized by forming two hydrogen bonds with the side chains of Arg-119^A^ and Asn-121^A^ (Fig. 4*B*). This newly established hydrogen-bonding network stabilizes the β5^A^-β6^A^ loop in the inactive conformation, which further induces the conformational changes of the structure elements at the heterodimer interface and the pseudo-allosteric site, including the N-terminal regions of the α7 helices in both the α and β subunits and the β5^B^-β6^B^ loop at the pseudo-allosteric site. Concurrently, some key hydrogen-bonding interactions observed in the α^Ca^β structure are disrupted, and several new hydrogen-bonding interactions are established to stabilize the conformations of these structure elements in a similar manner as in the α^Mg^γ structure (Fig. 4*B*). These results suggest that the inactive overall conformation of the α^NAD^β structure could be attributed to the NAD binding–induced conformational change of the β3^A^-α3^A^ loop at the active site, which stabilizes the side chain of Tyr-126^A^ and the β5^A^-β6^A^ loop in the inactive conformations.

### The active site in the αβ structures has a distorted geometry that is unfavorable for the metal ion binding

Our biochemical data show that the αβ heterodimer alone has a lower activity than the αγ heterodimer alone, and additionally the αβ heterodimer exhibits only slightly different *S*_0.5,ICT_ and *S*_0.5,NAD_ (about 2-fold differences) but a significantly (>20-fold) higher *S*_0.5,Mn_ compared with the αγ heterodimer (Table 1). These results suggest that the low activity of the αβ heterodimer alone might be due to the weak binding of the metal ion. As it is still unknown whether the αβ and αγ heterodimers could exist and function alone in the cells, we cannot rule out that the high *S*_0.5,Mn_ and low activity of the αβ heterodimer alone may be biologically irrelevant or are biochemical artifacts. Nonetheless, we performed a detailed structural comparison of the active sites in the αβ and αγ structures to explore the underlying mechanism.

**Figure 5.**
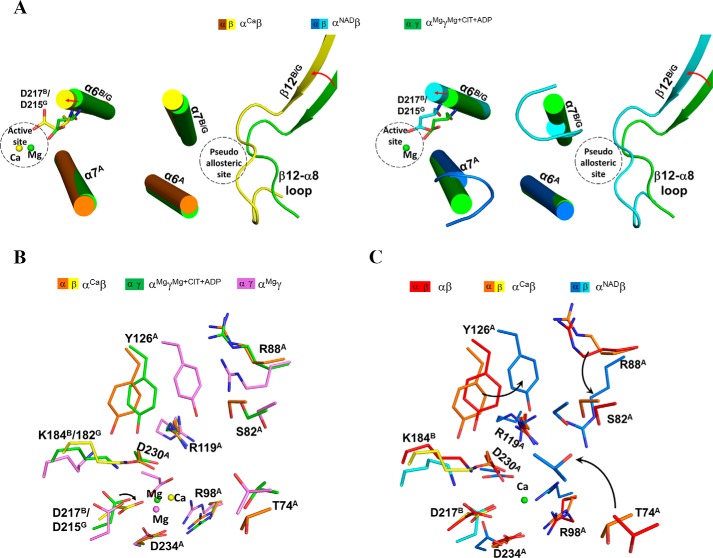
**The active site of the αβ heterodimer has a distorted geometry that is unfavorable for the metal ion binding.** *A*, conformational differences of the β12-α8 loop and the α6 and α7 helices at the heterodimer interface between α^Ca^β and α^Mg^γ^Mg+CIT+ADP^ (*left*) and between α^NAD^β and α^Mg^γ^Mg+CIT+ADP^ (*right*). The *color scheme* of the α^Ca^β, α^NAD^β, and α^Mg^γ^Mg+CIT+ADP^ structures is shown *above*. The β12^B^-α8^B^ loop in α^Ca^β and α^NAD^β is positioned much closer to the α6^A^ and α7^B^ helices at the heterodimer interface compared with that in α^Mg^γ^Mg+CIT+ADP^, which further causes conformational changes of the α6 and α7 helices toward the active site. *B*, structural comparison of the active sites in α^Ca^β, α^Mg^γ (*violet*), and α^Mg^γ^Mg+CIT+ADP^. The *color scheme* of the α^Ca^β, α^Mg^γ, and α^Mg^γ^Mg+CIT+ADP^ structures is shown *above. C*, structural comparison of the active sites in α^Ca^β, αβ (*red*), and α^NAD^β. The *color scheme* of the αβ, α^Ca^β, and α^NAD^β structures is shown *above*. Although most of the residues composing the active site are in proper positions to interact with NAD and ICT, Asp-230^A^, Asp-234^A^, and particularly Asp-217^B^ in the αβ structures are not in appropriate positions or conformations to bind the metal ion in a catalysis-relevant manner.

Our structural comparisons show that the β12^B^-α8^B^ and β3^B^-α3^B^ loops of the β subunit exhibit substantially different conformations from those of the γ subunit. Compared with the α^Mg^γ^CIT+Mg+ADP^ structure, the β12^B^-α8^B^ loop in the α^Ca^β and α^NAD^β structures is drawn closer to the heterodimer interface and makes interactions with the α7^B^ and α6^A^ helices (Fig. 5*A*). As a result, the α7^B^, α6^B^, and α6^A^ helices at the heterodimer interface are pushed slightly toward the active site, and several residues on these helices undergo conformational changes to varied extents (Fig. 5*A*). In particular, to avoid steric conflicts with the side chains of Asp-230^A^ and Asp-234^A^ on the α7^A^ helix, the side chain of Asp-217^B^ on the α6^B^ helix (equivalent to Asp-215^G^) is oriented toward the Ca^2+^-binding site and thus pushes the Ca^2+^ away by about 1.2 and 2 Å compared with the Mg^2+^ in the α^Mg^γ^Mg+CIT+ADP^ and α^Mg^γ structures, respectively (Fig. 5*B* and Fig. S4). These results indicate that in the α^Ca^β structure, the three conserved Asp residues (Asp-230^A^, Asp-234^A^, and particularly Asp-217^B^) are not in proper positions or conformations to bind the metal ion in a catalysis-relevant manner. Similarly, in the αβ structure, Asp-230^A^, Asp-234^A^, and Asp-217^B^ assume positions comparable with those in the α^Ca^β structure and are not in proper positions or conformations to bind the metal ion effectively (Fig. 5*C* and Fig. S4). In the α^NAD^β structure, due to the conformational changes of the large domain of the α subunit, several key residues, including Thr-74^A^, Ser-82^A^, Arg-88^A^, and Arg-98^A^, are drawn closer to the active site to interact with NAD; nonetheless, Asp-230^A^, Asp-234^A^, and Asp-217^B^ are also not in appropriate positions or conformations to bind the metal ion effectively (Fig. 5*C* and Fig. S4), which is consistent with the structural data that despite the presence of Ca^2+^, ICT, and NAD in the crystallization solution, only an NAD is bound at the active site. These findings suggest that owing to the conformational changes of the heterodimer interface, the active site of the αβ heterodimer has a distorted geometry, and particularly the three conserved Asp residues (Asp-230^A^, Asp-234^A^, and Asp-217^B^) are not in appropriate positions or conformations to bind the metal ion effectively or in a catalysis-relevant manner. This might explain why the αβ heterodimer alone has a high *S*_0.5,Mn_ and a low activity.

### NADH binds to the NAD-binding site and inhibits the activity of the αβ heterodimer

Our previous biochemical data show that NADH can inhibit the activities of both the αβ and αγ heterodimers (39). Our previous structural and biochemical studies of the αγ heterodimer also show that NADH can bind to the active site to prevent the NAD binding and meanwhile can also bind to the allosteric site to block the ADP and CIT binding, leading to the inhibition of the activity of the αγ heterodimer (41). Unlike the α^Mg+NADH^γ^NADH^ structure, in the α^NADH^β structure, NADH binds only to the active site and occupies the spatial position of NAD. The α^NADH^β structure is almost identical to the α^NAD^β structure (superposition of the two structures yields an RMSD of 0.5 Å for about 610 Cα atoms). The NADH binding induces almost identical conformational changes of the large domain of the α subunit as the NAD binding, and the NADH retains almost identical interactions with the surrounding residues as the NAD. These results indicate that NADH competes with NAD to bind to the active site of the αβ heterodimer and thus inhibits the activity of the αβ heterodimer.

## Discussion

Human NAD-IDH is a key enzyme in the TCA cycle, which catalyzes the oxidative decarboxylation of ICT into α-KG. The holoenzyme of human NAD-IDH is composed of the αβ and αγ heterodimers. Our previous biochemical studies demonstrated that the αγ heterodimer alone exhibits a high enzymatic activity and kinetic properties comparable with those of the holoenzyme and can be activated by CIT and ADP but inhibited by NADH; on the other hand, the αβ heterodimer alone exhibits a low activity and cannot be activated by the activators but can be inhibited by NADH (39). Our previous structural and kinetic studies together revealed the activation mechanism of the αγ heterodimer by CIT and ADP and the inhibition mechanism by NADH (39, 40). However, the molecular basis for the function of the αβ heterodimer is still unclear. In this work, we determined the crystal structures of the αβ heterodimer of human NAD-IDH with the α subunit in apo form, and in Ca^2+^-bound, NAD-bound, and NADH-bound forms. Analyses of those structures and their comparisons with the αγ structures reveal the conformational differences between the αβ and αγ heterodimers and provide the molecular basis for the function of the αβ heterodimer.

Our structural comparisons show that the αβ heterodimer has a similar yet more compact overall structure compared with the αγ heterodimer and contains a pseudo-allosteric site that exhibits substantial conformational differences from the allosteric site in the αγ heterodimer. In particular, the β12^B^-α8^B^ and β3^B^-α3^B^ loops at the pseudo-allosteric site adopt significantly different conformations from the corresponding loops at the allosteric site, which occupy parts of the ADP- and CIT-binding sites and thus prevent the binding of the activators, providing the molecular basis for why the αβ heterodimer cannot be regulated by the activators. In addition, our structural comparisons show that in all of the αβ structures, owing to the conformational changes of the β12^B^-α8^B^ loop and the α6 and α7 helices at the heterodimer interface, the active site assumes a distorted geometry, and the three conserved Asp residues (Asp-230^A^, Asp-234^A^, and particularly Asp-217^B^) are not in appropriate positions or conformations to bind the metal ion effectively or in a catalysis-relevant manner, which might explain why the αβ heterodimer alone has a high *S*_0.5,Mn_ and a low activity.

Our structural and biochemical studies demonstrate that the αγ heterodimer exhibits a high activity and contains a functional allosteric site that can bind the activators, whereas the αβ heterodimer exhibits a low activity and contains a pseudo-allosteric site that cannot bind the activators. The holoenzyme has a much higher activity than the sum of the activities of the two heterodimers, and in the holoenzyme, the α subunits in both the αβ and αγ heterodimers can be allosterically regulated by the γ subunit, and both heterodimers have a normal activity and contribute to the full activity of the holoenzyme (39). These results imply that in the holoenzyme, the αβ and αγ heterodimers can communicate with each other in a cooperative manner to ensure the full activity of the holoenzyme. These results also suggest that in the holoenzyme, the binding of the activators to the allosteric site in the γ subunit should induce conformational changes not only at the active site of the αγ heterodimer but also at the active site of the αβ heterodimer, and in addition, the αβ heterodimer is likely to exhibit some conformational changes different from those observed in the αβ heterodimer alone but similar to those observed in the αγ heterodimer; thus, the active site of the αβ heterodimer could assume a proper conformation that is suitable for the binding of the metal ion, substrate, and cofactor, leading the αβ heterodimer to exert a regular catalytic reaction and have a normal activity as the αγ heterodimer.

It is interesting to observe that the αβ and α^Ca^β structures assume an active overall conformation similar to that in the α^Mg^γ^Mg+CIT+ADP^ structure, whereas the α^NAD^β and α^NADH^β structures assume an inactive overall conformation similar to that in the α^Mg^γ structure. Structural analyses suggest that the active overall conformation of the αβ and α^Ca^β structures could be attributed to the changes of some residues at the pseudo-allosteric site and a unique hydrogen-bonding network, which stabilize the side chain of Tyr-137^B^ and the β5^B^-β6^B^ loop in the active conformations and further trigger the conformational changes of the heterodimer interface and the active site. On the other hand, the inactive overall conformation of the α^NAD^β and α^NADH^β structures could be attributed to the NAD binding–induced conformational change at the active site and a newly established hydrogen-bonding network, which stabilize the side chain of Tyr-126^A^ and the β5^A^-β6^A^ loop in the inactive conformations and further trigger the conformational changes of the heterodimer interface and the pseudo-allosteric site. However, in the αγ structures, the binding of the activators to the allosteric site seems to dictate the conformational changes of the allosteric site, the heterodimer interface, and the active site from the inactive conformations to the active conformations. In other words, the communication between the pseudo-allosteric site and the active site in the αβ heterodimer is bidirectional and the conformational changes can be triggered from either side, whereas the communication between the allosteric site and the active site in the αγ heterodimer is unidirectional and the conformational changes are always triggered from the allosteric site to the active site. Nonetheless, it is unclear whether NAD binding to the αγ heterodimer might induce similar conformational changes from the active site to the allosteric site.

Our structural studies show that in all of the αβ and αγ structures, the two heterodimers always form a dimer of heterodimers or a heterotetramer via the clasp domains, and the structure elements at the heterodimer and heterodimer-heterodimer interfaces assume similar structures and maintain similar interactions (Fig. S5). In the structure of yeast NAD-IDH, two heterodimers are assembled into a dimer of heterodimers or a heterotetramer via the clasp domains in a similar manner as the αβ and αγ heterodimers (30). These results lead us to hypothesize that the αβ and αγ heterodimers may interact with each other via the clasp domains to assemble into the α_2_βγ heterotetramer, and the clasp domains at the heterodimer-heterodimer interface may help to mediate the transmission of the activation signal from the allosteric site in the γ subunit to the active sites of the α subunits in both the αβ and αγ heterodimers, achieving the full activity of the α_2_βγ heterotetramer or the holoenzyme. Further structural studies of the α_2_βγ heterotetramer will resolve these issues and eventually uncover the exact functions and the molecular mechanisms for the cooperativity and allosteric regulation of the αβ and αγ heterodimers in the holoenzyme.

## Materials and methods

### Cloning, expression, and purification

The WT and mutant αβ heterodimers of human NAD-IDH were prepared using the method described previously (39). Briefly, the DNA fragments encoding the α and β subunits of human NAD-IDH were cloned into the co-expression vector pQlinkN with the C termini of the β subunit attached with a TEV protease cleavage site and a His_6_ tag to construct the pQlinkN-α-β-tev-His_6_ plasmid. The plasmid was transformed into *E. coli* BL21 (DE3) Codon-Plus strain (Novagen). When the culture of the transformed cells reached an *A*_600_ of 0.5, the protein expression was induced by 0.4 mm isopropyl 1-thio-β-d-galactopyranoside for 20 h at 25 °C. The bacterial cells were harvested, resuspended, and sonicated on ice in the lysis buffer (50 mm HEPES (pH 7.4), 200 mm NaCl, 0.2 mm MnCl_2_, 10% (w/v) glycerol, and 7.2 mm β-mercaptoethanol) supplemented with 1 mm phenylmethylsulfonyl fluoride. The target protein was purified by affinity chromatography using a nickel-nitrilotriacetic acid column (Qiagen) with the lysis buffer supplemented with 20 and 200 mm imidazole serving as the washing and elution buffers, respectively. The elution fraction was dialyzed overnight against the lysis buffer supplemented with the proper amount of TEV protease (about 40 μg/ml) to decrease the concentration of imidazole and to cleave the His_6_ tag of the target protein. Thus, the C terminus of the β subunit contains a few extra residues (ENLYFQ) from the TEV cleavage site. The cleavage mixture was reloaded on a nickel-nitrilotriacetic acid column and washed with the lysis buffer supplemented with 10 mm imidazole. The flow-through fraction contains the target protein, which was further purified by gel filtration using a Superdex 200 10/300 GL column (GE Healthcare) equilibrated with the storage buffer (10 mm HEPES, pH 7.4, 200 mm NaCl, and 5 mm β-mercaptoethanol). Purity of the protein was assessed by 12% SDS-PAGE. The purified proteins were concentrated to 10 mg/ml for structural and biochemical studies.

### Crystallization, diffraction data collection, and structure determination

Crystallization was performed using the hanging-drop vapor diffusion method at 20 °C by mixing equal volume (1 μl) of protein solution and reservoir solution. Crystals of the αβ heterodimer were obtained at two different crystallization conditions. Crystals of the αβ heterodimer with the α subunit in apo form (αβ) grew at condition I containing 8% (v/v) tacsimate (pH 6.0) and 20% (w/v) PEG 3350. Crystals of the αβ heterodimer with the α subunit bound with a Ca^2+^ at the active site (α^Ca^β), bound with an NAD at the active site (α^NAD^β), and bound with an NADH at the active site (α^NADH^β) all grew at condition II containing 0.2 m calcium acetate (pH 7.5) and 20% (w/v) PEG 3350. Prior to diffraction data collection, crystals were cryoprotected using the reservoir solution supplemented with 25% ethylene glycol. Diffraction data were collected from crystals at 100 K at BL17U1 of the Shanghai Synchrotron Radiation Facility or BL18U1 and BL19U1 of the National Facility for Protein Science Shanghai and processed with HKL3000 (43). Statistics of the diffraction data are summarized in Table 2.

The αβ structure was solved with the molecular replacement method as implemented in the program Phaser (44) using the α^Mg^γ structure (Protein Data Bank code 5GRH) (40) as the search model, which was then used as the search model to solve the α^Ca^β, α^NAD^β, and α^NADH^β structures. Initial structure refinement was carried out with the program Phenix (45), and final structure refinement was performed with the program REFMAC5 (46). Model building was performed with the program Coot (47). Stereochemistry and quality of the structure models were analyzed using programs in the CCP4 suite (48) and the PISA server (49). The structure figures were prepared using PyMOL (50), and the structure-based sequence alignment figures were prepared using ESPpript 3.0 (51). Statistics of the structure refinement and the final structure models are also summarized in Table 2.

### Enzymatic activity assay

Enzymatic activities of the WT and mutant αβ heterodimers were determined at 25 °C using the method described previously (39). The standard reaction solution (1 ml) consisted of 2 ng/ml enzyme, 33 mm Tris acetate (pH 7.4), 40 mm ICT, 2 mm Mn^2+^, and 3.2 mm NAD. The activity is defined as the amount of NADH produced per minute per milligram of the enzyme. Kinetic parameters of the enzyme in the absence of activators were determined at standard conditions with varied concentrations of ICT (0–40 mm), Mn^2+^ (0–10 mm), or NAD (0–10 mm) to obtain the *V*_max_ and the *S*_0.5_ for ICT, Mn^2+^, or NAD, respectively. Kinetic parameters in the presence of activators were determined at the same conditions containing 1 mm CIT and/or 1 mm ADP. The initial rate was determined from the slope of a linear fit of the early time point data. The kinetic parameters were obtained by fitting the experimental data into the Michaelis–Menten equation, *V* = *V*_max_ × [S]/(*S*_0.5_ + [S]), using the program Graphpad Prism (GraphPad Software), where *S*_0.5_ represents the apparent *K_m_* (the substrate concentration at the reaction velocity of 0.5 × *V*_max_), and [S] is the concentration of ICT, Mn^2+^, or NAD. All experiments were performed in at least two independent measurements, and the values were the averages of the measurements with the S.E.

### Data accession

The αβ, α^Ca^β, α^NAD^β, and α^NADH^β structures of human NAD-IDH have been deposited in the Protein Data Bank with accession codes 6KDF, 6KDE, 6KDY, and 6KE3, respectively.

## Author contributions

P. S., T. M., and J. D. formal analysis; P. S. validation; P. S., T. M., T. Z., H. Z., J. Z., and Y. L. investigation; P. S. and J. D. writing-original draft; J. D. conceptualization; J. D. supervision; J. D. funding acquisition; J. D. writing-review and editing.

## Supplementary Material

Supporting Information

## Footnotes

2 The abbreviations used are:

TCA: tricarboxylic acid
IDH: isocitrate dehydrogenase
NAD-IDH: NAD-dependent IDH
NADP-IDH: NADP-dependent IDH
RMSD: root mean square deviation
ICT: isocitrate
α-KG: α-ketoglutarate
TEV: tobacco etch virus
CIT: citrate.
