# Supplementary material for: Molecular basis for the function of the αβ heterodimer of human NAD-dependent isocitrate dehydrogenase
Source: J Biol Chem. 2019 Sep 12;294(44):16214–27. doi: 10.1074/jbc.RA119.010099 (PMC6827300; doi:10.1074/jbc.RA119.010099)
Supplement: Supporting Information [file supp_294_44_16214__index.html]

Molecular basis for the function of the αβ heterodimer of human NAD-dependent isocitrate dehydrogenase — Crystal structure of the αβ heterodimer of human NAD-IDH — Molecular basis for the function of the αβ heterodimer of human NAD-dependent isocitrate dehydrogenase — Crystal structure of the αβ heterodimer of human NAD-IDH — Supporting Information 

# Molecular basis for the function of the αβ heterodimer of human NAD-dependent isocitrate dehydrogenase

## Supporting Information

- Supporting Information (to be published online) - Supplementary Information
