# Supplementary material for: Molecular basis for the function of the αβ heterodimer of human NAD-dependent isocitrate dehydrogenase
Source: J Biol Chem. 2019 Sep 12;294(44):16214–27. doi: 10.1074/jbc.RA119.010099 (PMC6827300; doi:10.1074/jbc.RA119.010099)

Figure S1. Sequence alignments of NAD-IDHs (IDH3s) from representative vertebrates. (A) Sequence alignment of the α, β, and γ subunits of HsIDH3. (B) Sequence alignment of the α subunits of IDH3s from representative vertebrates. (C) Sequence alignment of the β subunits of IDH3s from representative vertebrates. (D) Sequence alignment of the γ subunits of IDH3s from representative vertebrates. The abbreviations are as follows: HsIDH3, *Homo sapien* IDH3; RnIDH3, *Rattus norvegicus* IDH3; AmIDH3, *Ailuropoda melanoleuca* IDH3; AcIDH3, *Anoils carolinensis* IDH3; DrIDH3, *Danio rerio* IDH3; and XlIDH3, *Xenopus laevis* IDH3. The secondary structures of the α, β, and γ subunits of HsIDH3 are placed on the top of the alignments. The β3-α3 and β12-α8 loops are highlighted with green boxes. The C-terminal region of the β subunit which was substituted with the equivalent of the α subunit is highlighted with blue boxes.

A

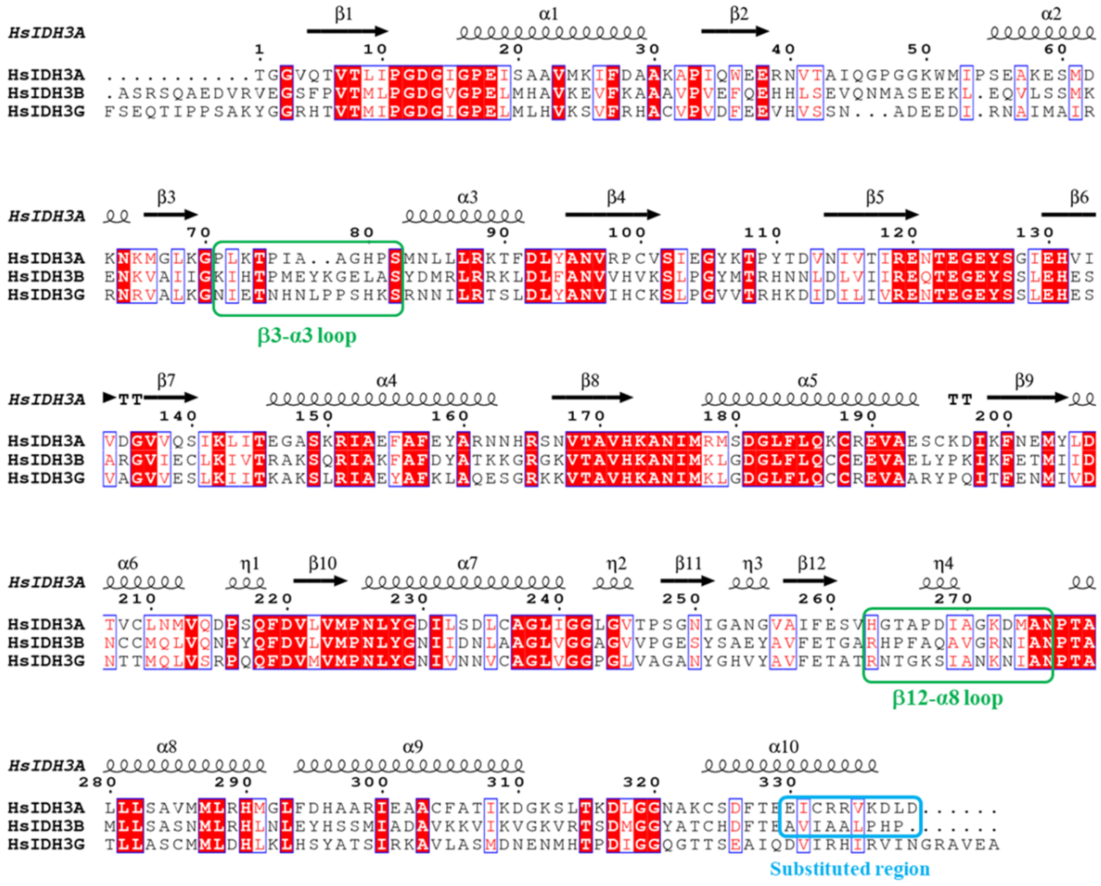

B

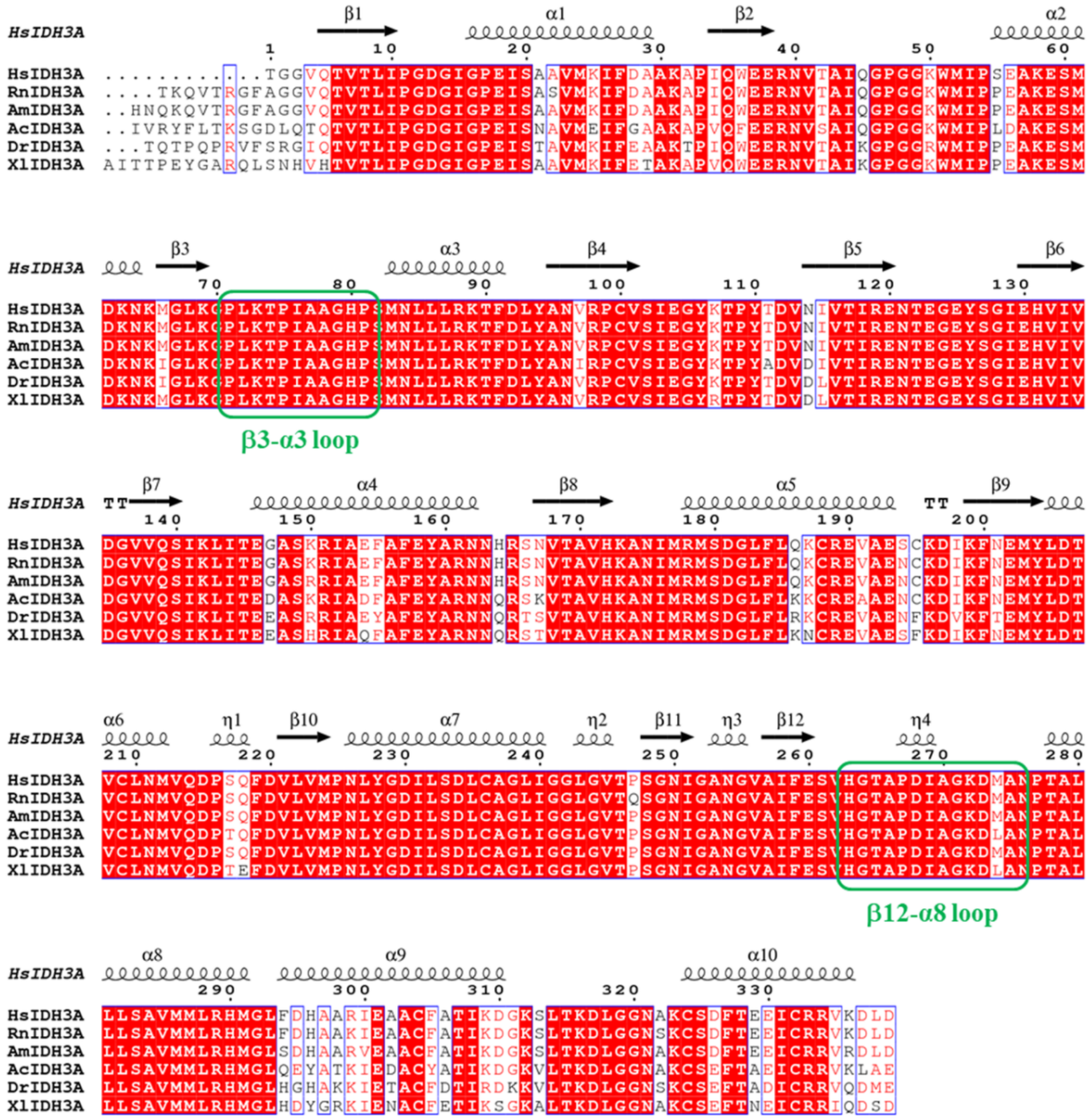
HsIDH3A
HsIDH3A TGGVQTVTLIPGDGIGPEISAAVMKIFDAAKAPIQWEERNVTAIQGPGGKWMIPSEAKESM
RnIDH3A TKQVTRGFAGGVQTVTLIPGDGIGPEISASVMKIFDAAKAPIQWEERNVTAIQGPGGKWMIPPEAKESM
AmIDH3A HNQKQVTRGFAGGVQTVTLIPGDGIGPEISAAVMKIFDAAKAPIQWEERNVTAIQGPGGKWMIPPEAKESM
AcIDH3A IVRYFLTKSGDLQTQTVTLIPGDGIGPEISNAVMEIFGAAKAPVQFEERNVSAIQGPGGKWMIPLDAKESM
DrIDH3A TQTPQPRVFSRGIQTVTLIPGDGIGPEISTAVMKIFEAAKTPIQWEERNVTAIKGPGGRWMIPPEAKESM
XlIDH3A AITTPEYGARQLSNHVHTVTLIPGDGIGPEISAAVMKIFETAKAPVQWEERNVTAIKGPGGKWMIPPEAKESM
HsIDH3A DKNKMGLKGPLKTPIAAGHPSMNLLLRKTFDLYANVRPCVSIEGYKTPYTDVNIVTIRENTEGEYSGIEHVIV
RnIDH3A DKNKMGLKGPLKTPIAAGHPSMNLLLRKTFDLYANVRPCVSIEGYKTPYTDVNIVTIRENTEGEYSGIEHVIV
AmIDH3A DKNKMGLKGPLKTPIAAGHPSMNLLLRKTFDLYANVRPCVSIEGYKTPYTDVNIVTIRENTEGEYSGIEHVIV
AcIDH3A DKNKIGLKGPLKTPIAAGHPSMNLLLRKTFDLYANIRPCVSIEGYKTPYADVDIVTIRENTEGEYSGIEHVIV
DrIDH3A DKNKIGLKGPLKTPIAAGHPSMNLLLRKTFDLYANVRPCVSIEGYKTPYTDVDLVTIRENTEGEYSGIEHVIV
XlIDH3A DKNKMGLKGPLKTPIAAGHPSMNLLLRKTFDLYANVRPCVSIEGYRTPYTDVDLVTIRENTEGEYSGIEHVIV
β3-α3 loop
HsIDH3A DGVVQSIKLITEGASKRIAEFAFEYARNNHRSNVTAVHKANIMRMSDGLFLQKCREVAESCKDIKFNEMYLDT
RnIDH3A DGVVQSIKLITEGASKRIAEFAFEYARNNHRSNVTAVHKANIMRMSDGLFLQKCREVAENCKDIKFNEMYLDT
AmIDH3A DGVVQSIKLITEGASRRIAEFAFEYARNNHRSNVTAVHKANIMRMSDGLFLQKCREVAENCKDIKFNEMYLDT
AcIDH3A DGVVQSIKLITEDASKRIADFAFEYARNNQRSKVTAVHKANIMRMSDGLFLKKCREAAENCKDIKFNEMYLDT
DrIDH3A DGVVQSIKLITEEASRRIAEYAFEYARNNQRTSVTAVHKANIMRMSDGLFLRKCREVAENFKDVKFTEMYLDT
XlIDH3A DGVVQSIKLITEEASHRIAQFAFEYARNNQRSTVTAVHKANIMRMSDGLFLKNCREVAESFKDIKFNEMYLDT
HsIDH3A VCLNMVQDPSQFDVLVMPNLYGDILSDLCAGLIGGLGVTPSGNIGANGVAIFESVHGTAPDIAGKDMANPTAL
RnIDH3A VCLNMVQDPSQFDVLVMPNLYGDILSDLCAGLIGGLGVTQSGNIGANGVAIFESVHGTAPDIAGKDMANPTAL
AmIDH3A VCLNMVQDPSQFDVLVMPNLYGDILSDLCAGLIGGLGVTPSGNIGANGVAIFESVHGTAPDIAGKDMANPTAL
AcIDH3A VCLNMVQDPTQFDVLVMPNLYGDILSDLCAGLIGGLGVTPSGNIGANGVAIFESVHGTAPDIAGKDLANPTAL
DrIDH3A VCLNMVQDPSQFDVLVMPNLYGDILSDLCAGLIGGLGVTPSGNIGANGVAIFESVHGTAPDIAGKDMANPTAL
XlIDH3A VCLNMVQDPTEFDVLVMPNLYGDILSDLCAGLIGGLGVTPSGNIGANGVAIFESVHGTAPDIAGKDLANPTAL
β12-α8 loop
HsIDH3A LLSAVMMLRHMGLFDHAARIEAACFATIKDGKSLTKDLGGNAKCSDFTEEICRRVKDLD
RnIDH3A LLSAVMMLRHMGLFDHAAKIEAACFATIKDGKSLTKDLGGNSKCSDFTEEICRRVKDLD
AmIDH3A LLSAVMMLRHMGLSDHAARVEAACFATIKDGKSLTKDLGGNAKCSDFTEEICRRVRDLD
AcIDH3A LLSAVMMLRHMGLQEYATKIEDACYATIKDGKVLTKDLGGNAKCSEFTAEICRRVKLAE
DrIDH3A LLSAVMMLRHMGLHGHAKKIETACFDTIRDKKVLTKDLGGNSKCSEFTADICRRVQDME
XlIDH3A LLSAVMMLRHMGLHDYGRKIENACFETIKSGKALTKDLGGNAKCSEFTNEICRRIQDSD

C

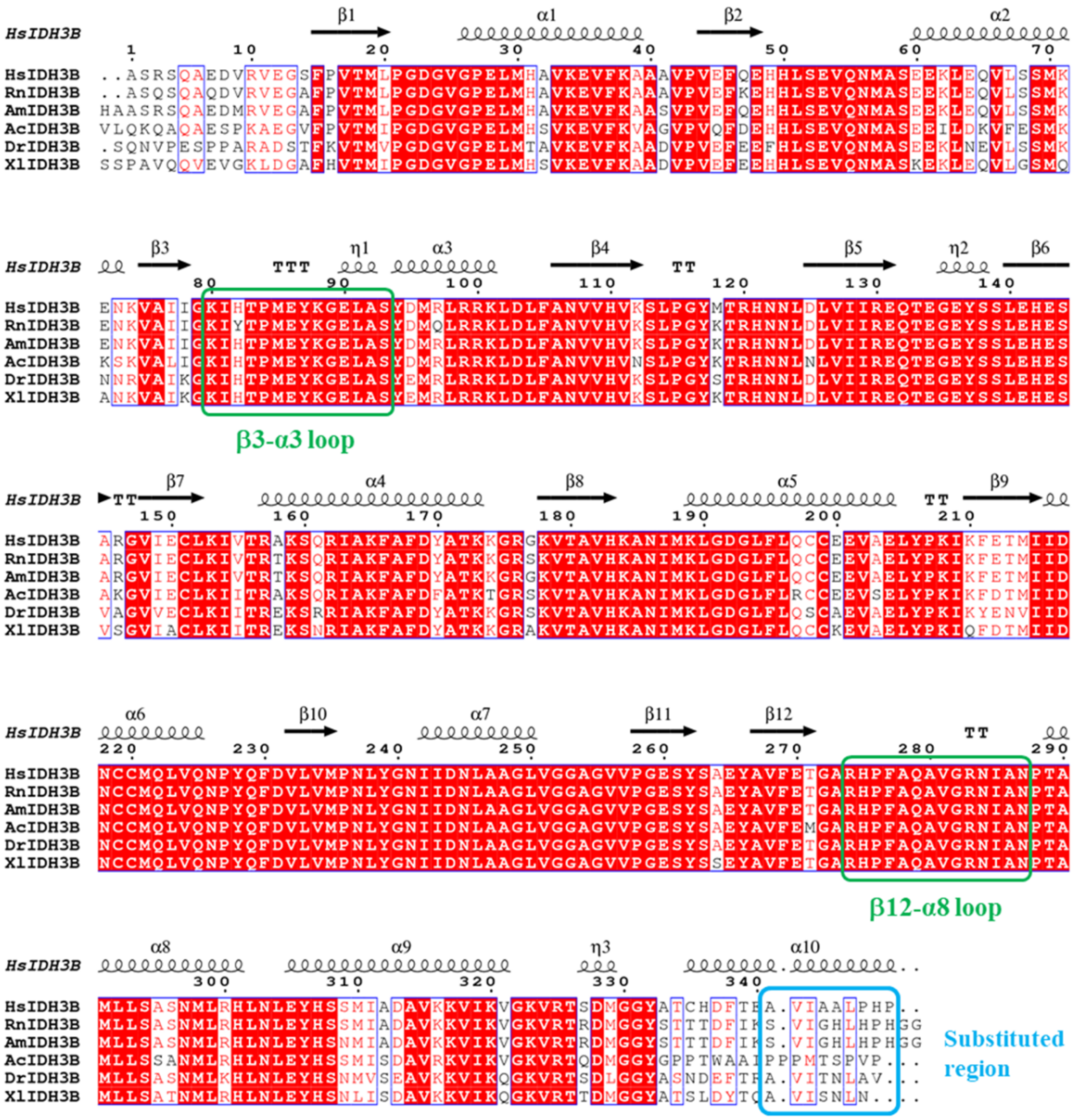

D

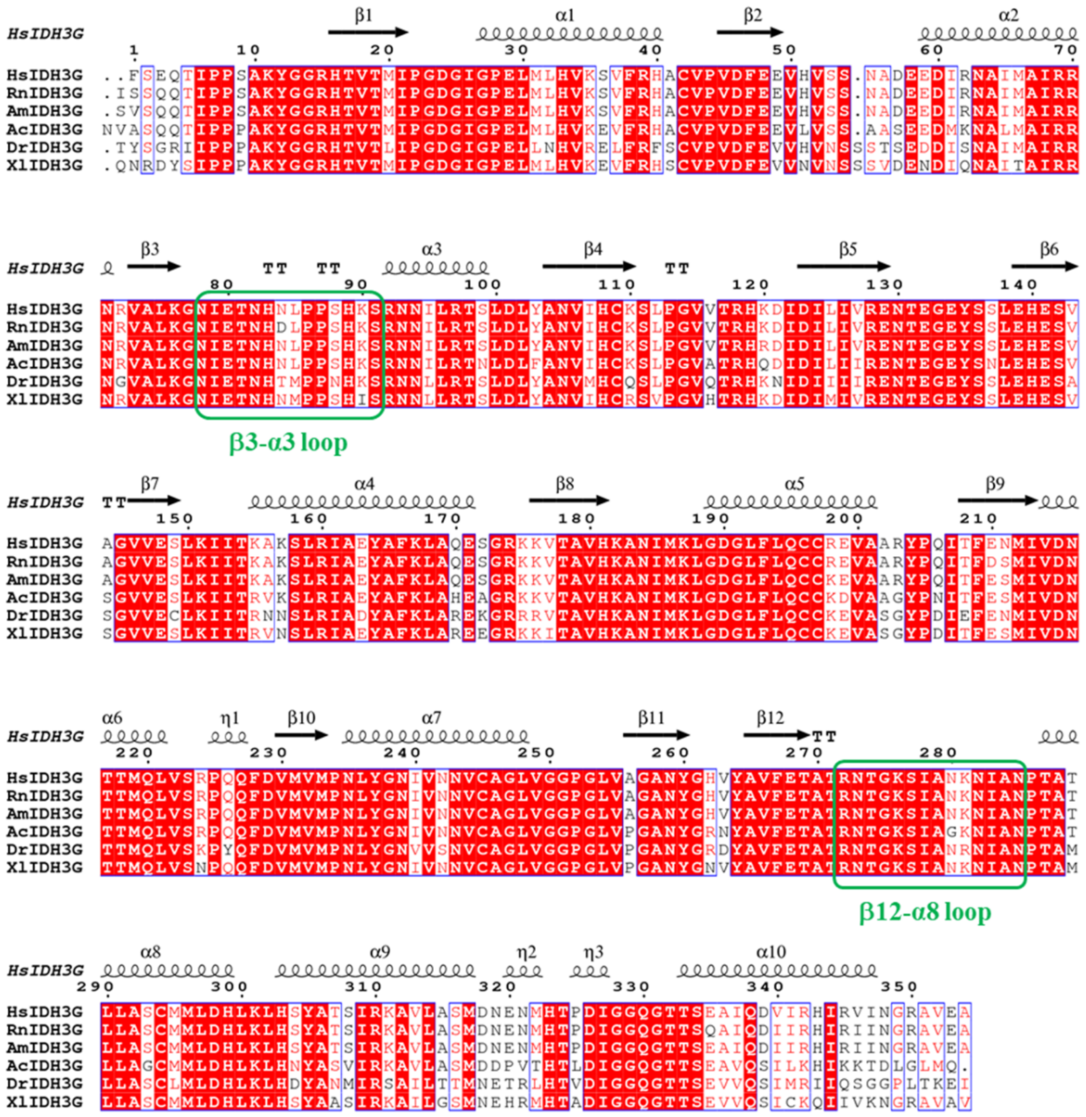
HsIDH3G
RnIDH3G
AmIDH3G
AcIDH3G
DrIDH3G
XlIDH3G
β3-α3 loop
β12-α8 loop

Figure S2. SEC and SDS-PAGE analyses of the purified wild-type (WT) and mutant (Mut) αβ heterodimers of human NAD-IDH. (A) Size exclusion chromatography (SEC) analyses of the WT and Mut αβ heterodimers. The WT and Mut αβ heterodimers show an elution peak at about 13.7 ml corresponding to an average molecular mass of approximately 80 kDa. (B) SDS-PAGE (12%) analyses of the WT and Mut αβ heterodimers with Coomassie blue staining. M: molecular mass standards. WT1: the elution fraction of the WT αβ heterodimer purified by affinity chromatography using a Ni-NTA column (Qiagen). MUT1: the elution fraction of the Mut αβ heterodimer purified by affinity chromatography using a Ni-NTA column. WT2: the elution fraction of the WT αβ heterodimer purified by SEC using a Superdex 200 10/300 GL column (GE Healthcare). MUT2: the elution fraction of the Mut αβ heterodimer purified by SEC using a Superdex 200 10/300 GL column. Control: the previously purified WT αβ heterodimer. The upper band represents the β subunit (39 kDa), and the lower band represents the α subunit (37 kDa).

A

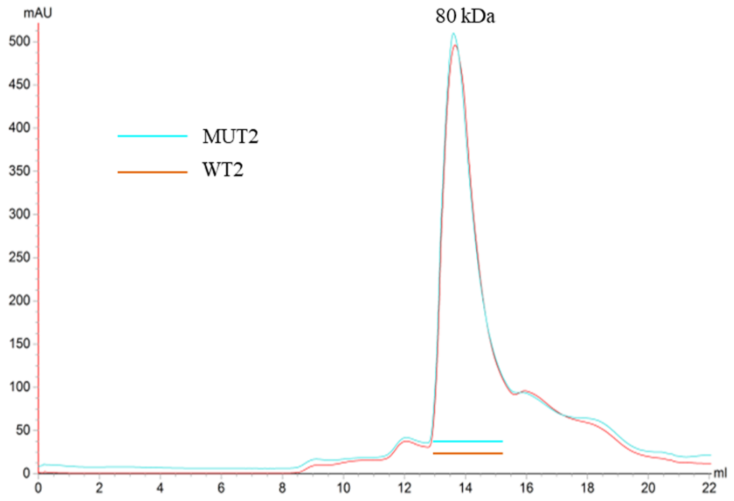

B

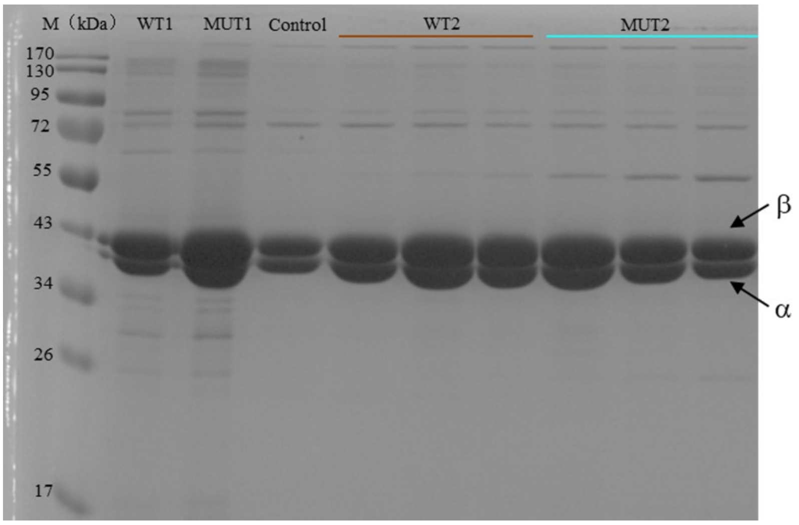

Figure S3. Portions of representative simulated annealing composite 2Fo-Fc omit maps ($\sigma = 1.0$). (A) The metal ion-binding site in the $\alpha^{Ca}\beta$ structure. (B) The bound NAD in the $\alpha^{NAD}\beta$ structure. (C) The bound NADH in the $\alpha^{NADH}\beta$ structure.

A **B** **C**

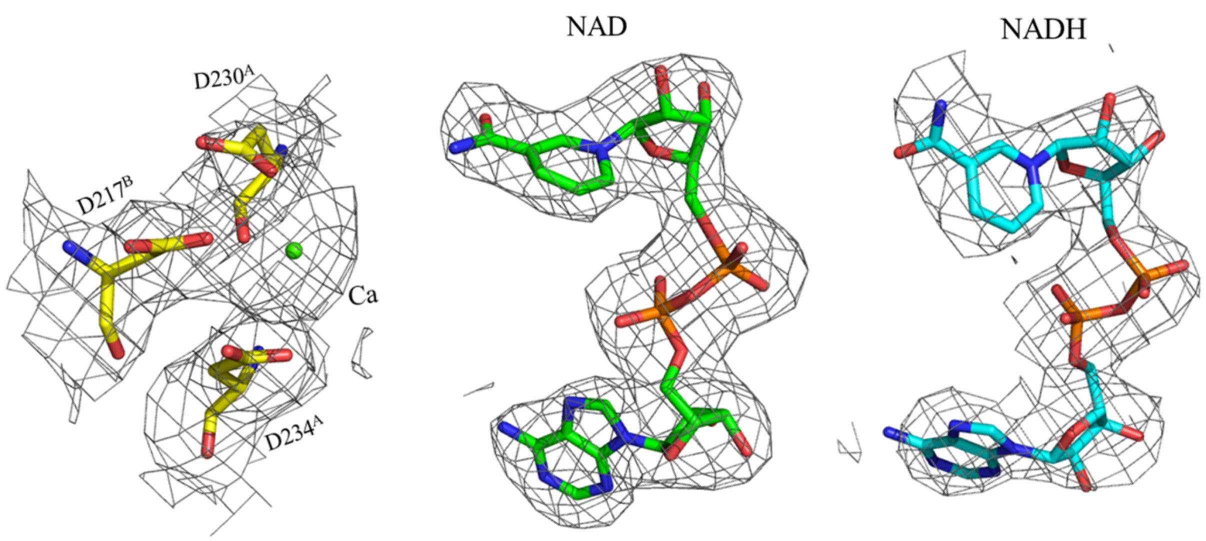

Figure S4. Comparison of the conformations of the three conserved Asp residues (Asp230^{A}, Asp234^{A}, and Asp217^{B} or Asp215^{G}) at the active sites of the αβ and αγ structures. (A) Conformations of the three Asp residues in the αβ, $\alpha^{Ca}\beta$, $\alpha^{NAD}\beta$, $\alpha^{Mg}\gamma$, and $\alpha^{Mg}\gamma^{Mg+CIT+ADP}$ structures. The distances between the metal ion and the three Asp residues are indicated. (B) Comparison of the conformations of the three Asp residues at the active sites of different αβ and αγ structures. The color-coding scheme of each structure is the same as in (A).

A

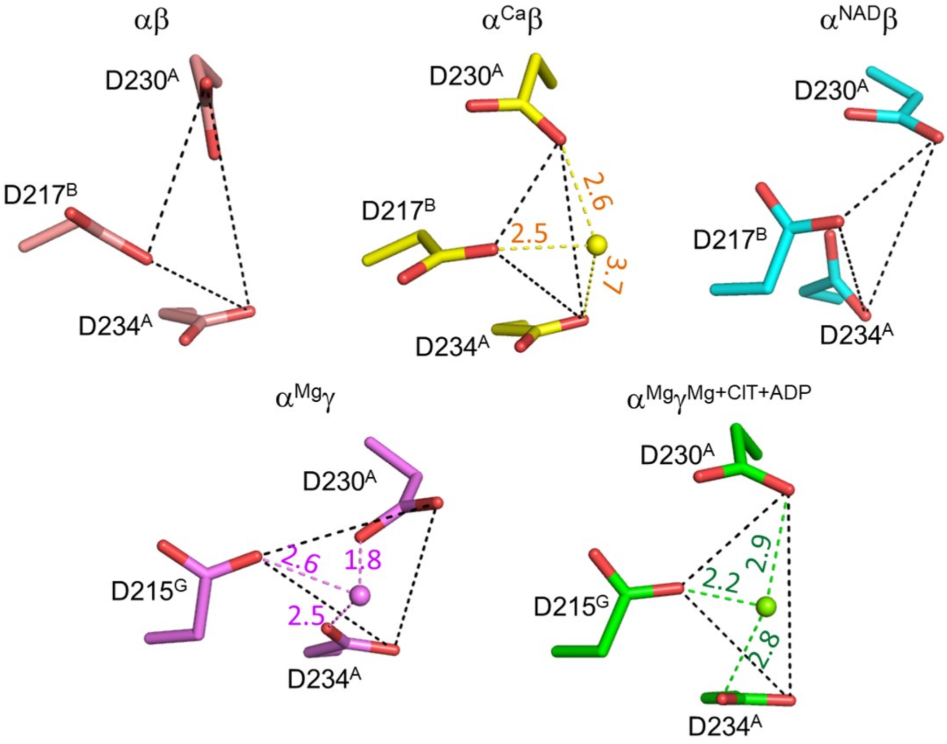

B

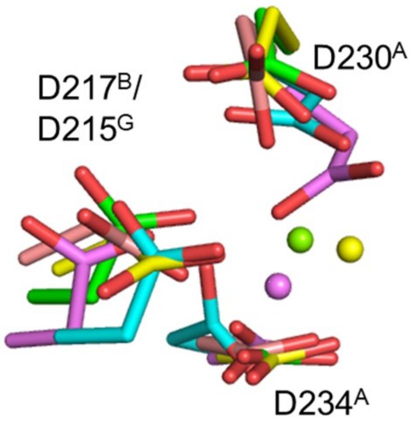

Figure S5. Overall structures of dimers of the $\alpha^{Ca}\beta$ heterodimers and the $\alpha^{Mg}\gamma$ heterodimers. (A) Overall structure of a dimer of the $\alpha^{Ca}\beta$ heterodimers in one asymmetric unit related by a non-crystallographic two-fold axis. The α and β subunits in the $\alpha^{Ca}\beta$ heterodimer are colored in yellow and cyan, respectively. The heterodimer-heterodimer interface is mediated by the clasp domains which are highlighted by the black box. (B) Overall structure of a dimer of the $\alpha^{Mg}\gamma$ heterodimers related by a crystallographic two-fold axis. The α and γ subunits in the $\alpha^{Mg}\gamma$ heterodimer are colored in salmon and slate, respectively. The heterodimer-heterodimer interface is mediated by the clasp domains which are highlighted by the black box.

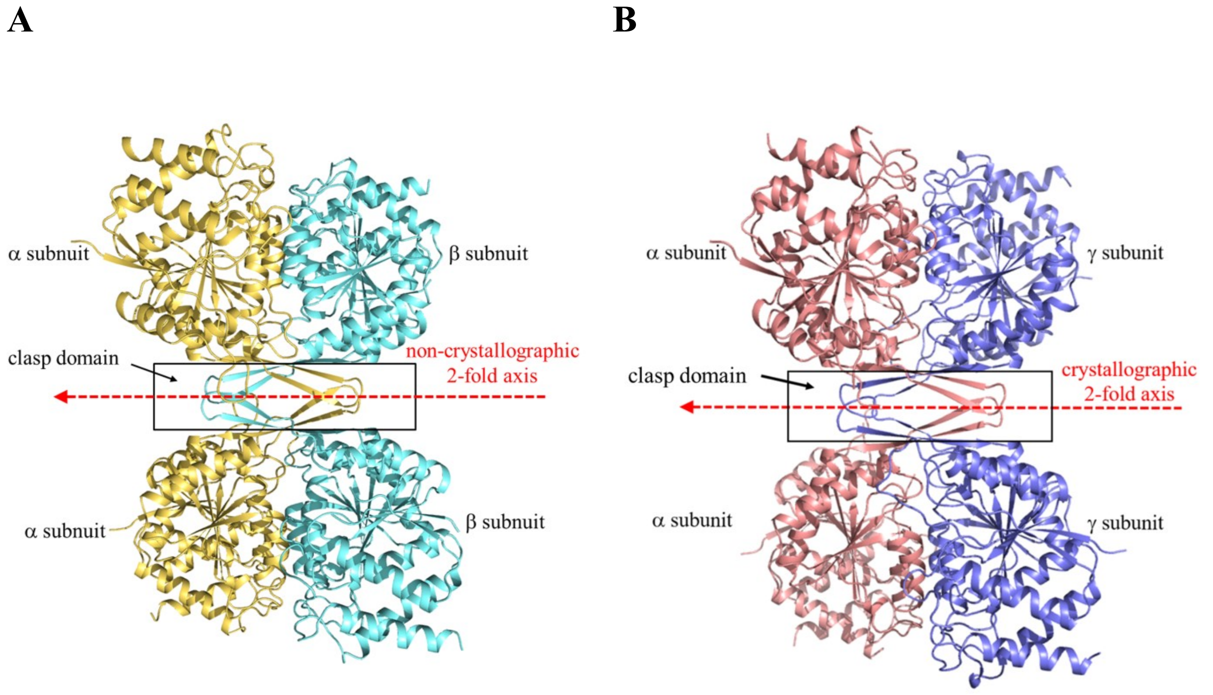

Supplement: Supporting Information [file supp_RA119.010099_154171_1_supp_382014_pwkmg2.pdf]
